# Glycemia and associated factors in a pediatric population in Mexico

**DOI:** 10.3389/fped.2023.1172837

**Published:** 2023-05-17

**Authors:** E. Lares-Villaseñor, S. Salazar-García, P. E. Cossío-Torres, D. L. Medina-Jasso, C. Aradillas-García, D. P. Portales-Pérez, J. M. Vargas-Morales

**Affiliations:** ^1^ Laboratorio de Análisis Clínicos, Facultad de Ciencias Químicas, Universidad Autónoma de San Luis Potosí, San Luis Potosi, Mexico; ^2^ Departamento de Salud Pública, Facultad de Medicina, Universidad Autónoma de San Luis Potosí, San Luis Potosi, Mexico; ^3^ Centro de Investigación Aplicada en Ambiente y Salud (CIAAS), Facultad de Medicina, Universidad Autónoma de San Luis Potosí, San Luis Potosi, Mexico; ^4^ Centro de Investigación en Ciencias de la Salud y Biomedicina (CICSaB), Facultad de Ciencias Químicas, Universidad Autónoma de San Luis Potosí, San Luis Potosi, Mexico

**Keywords:** glycemia, adolescents, risk factor, factors, children

## Abstract

**Aims:**

In this study, we evaluated the association of sociodemographic, lifestyle and cardiometabolic factors with blood glucose levels in children and adolescents in Mexico.

**Methods:**

An analytical cross-sectional study of 642 children and adolescents aged 6 to 19 years from different educational centers located in municipalities of the state of San Luis Potosí, Mexico, was carried out. Pearson *χ*^2^ and Spearman correlation tests and multiple linear regression models were used to evaluate the associations of the variables with glycemia.

**Results:**

The prevalence of prediabetes was 8.0% in both sexes. Male participants were more likely to develop hyperglycemia than female participants (OR 2.7, 95% CI: 1.5–5.0). The variables associated with glucose levels were male sex, high socioeconomic status, inadequate diet, high blood pressure, and increased total cholesterol, LDL cholesterol, and triglycerides, which also explained up to 15.6% (*p* < 0.05) of the variability in glucose concentrations.

**Conclusion:**

The detection of sociodemographic, lifestyle and cardiometabolic factors in children and adolescents will contribute to the implementation of prevention strategies for cardiometabolic diseases, among which prediabetes is common.

## Highlights

• Sociodemographic, lifestyle and cardiometabolic factors were related to glycemia.
• A total of 29.7% of the participants slept less than 8 h a day.
• The prevalence of prediabetes was 8% in children and adolescents in Mexico.
• Male participants were more likely to develop hyperglycemia than female participants.
• Sociodemographic and cardiometabolic factors explained 15.6% of the variability in both sexes and 17.3% of the variability in males.

## 1. Introduction

Noncommunicable chronic diseases (NCDs) are characterized by a slow progression, a long duration, and a high mortality rate resulting from a combination of different factors, including biological, environmental, social, economic and lifestyle factors (1, 2). Among the main NCDs are cardiovascular diseases (CVDs), chronic respiratory diseases, cancer and type 2 diabetes mellitus (T2D). Specifically, in Mexico, CVDs and T2D have been the leading causes of death since 2010–2017, as reported by the General Directorate of Epidemiology (3). However, the increase in obesity and overweight in Mexican adolescents and children has been evident in recent years (4), with more than 30% being overweight or obese while also experiencing comorbidities such as prehypertension, insulin resistance (IR) and prediabetes (5, 6).

Prediabetes is considered a risk factor for T2D and CVDs, which are also considered cardiometabolic diseases (7). However, its early detection is difficult in some cases because some people do not present obvious symptoms or signs until they develop T2D. For example, 88 million American adults have prediabetes, but more than 80% of them do not know they have it (8). In Mexico, the prevalence of prediabetes was greater than 13% in subjects under 20 years of age according to one study (9). However, current epidemiological data on the prevalence of NCDs, such as prediabetes, in Mexican children and adolescents are limited. In addition, it is important to highlight the lack of information on the influence of factors other than behavioral factors, such as social, economic or biological factors, which make the problem more complex. Therefore, the evaluation of different influencing factors of glucose concentrations would provide more information on the possible interrelated causes of prediabetes, thereby contributing to a better understanding of the development of NCDs with an emphasis on hyperglycemia as a condition for which school children have greater risk. In consideration of the aforementioned concerns, the objective of this study was to evaluate the association of sociodemographic, lifestyle and cardiometabolic factors with glucose levels in children and adolescents in Mexico.

## 2. Material and methods

### 2.1. Study design and participants

An analytical cross-sectional study using stratified sampling was performed by selecting participants from different educational centers (elementary and high school) located in Ahualulco, Moctezuma, San Luis Potosí, Matehuala and Villa de Guadalupe, which are municipalities in the state of San Luis Potosí, Mexico. The data were collected during 2015–2016. A total of 955 Mexican volunteers between 6 and 19 years of age were selected; however, only 642 had the authorization of their parent or guardian and a letter of consent as well as complete information for the following categories of variables: sociodemographic factors (age, sex and socioeconomic status); lifestyle factors [time spent in hours in front of electronic screens (cell phone, video games and TV), exercise, hours of sleep and diet]; and cardiometabolic factors (anthropometric variables, systolic and diastolic blood pressure and biochemical variables). The study followed the guidelines of the Declaration of Helsinki and was approved by the Comité Estatal de Ética en Investigación en Salud del Estado de San Luis Potosí, México SLP/003-2015.

### 2.2. Assessment of sociodemographic variables

The information was collected using questionnaires that were answered by the volunteers together with their parents or guardian. Information such as age and sex was collected. Socioeconomic status was evaluated using the questionnaire standardized for the Mexican population by the Mexican Association of Opinion and Market Intelligence Agencies (AMAI), which is based on scores that classify families based on the level of income and satisfaction of basic household needs, specifically, food, footwear, housing and utilities. In brief, the scores are classified into 7 categories, and the A/B category (planning and future) classifies households where the parents can afford to take international vacations or own their own businesses. The C+ category (entertainment and communication) classifies households where the children attend private schools or the parents are professionals or managers of a company. C (practical life) classifies households with a basic entertainment infrastructure, and their main expenditure is the children's education. The C- category (minimum practical conditions) are those households that have a fixed internet connection, and approximately 40% of their expenditures are food and 5% include clothing and footwear.

The D+ category (basic sanitary conditions) includes households with minimal sanitation infrastructure and children attending public schools. The D category (walls and some services) classifies households where 46% of their expenditures is food and they lack the majority of utilities. The E category (shortage) includes households that lack all utilities and their income is not sufficient for food (10).

### 2.3. Assessment of lifestyle variables

Questionnaires were used to collect information on the amount of time in hours per day that volunteers spent in front of electronic screens. In addition, a questionnaire was conducted to determine the minutes per week that the subjects devoted to vigorous-intensity aerobic activities (such as playing basketball or soccer); an adequate exercise pattern was defined as vigorous-intensity aerobic activities carried out for 60 min a day for at least three days a week according to the recommendations made by the World Health Organization (WHO) (11). We followed the recommendations of the National Sleep Foundation to assess sleep conditions (12), where an average of 8 h a day was considered good quality sleep. Finally, diet was evaluated based on a questionnaire adapted and validated for the Mexican population for which a healthy eating index (HEI) was used and the score is proportional to the consumption of a healthy diet (13).

### 2.4. Assessment of cardiometabolic variables

#### 2.4.1. Anthropometric measurements

Height was assessed with a SECA ® stadiometer (Seca 213, Seca, Hanover, MD, USA, 2009) (14). Weight was measured with a calibrated electronic monitor TANITA®UM-081 (Tanita UM-081, Tokyo, Japan). BMI was calculated with the formula BMI = weight/height [kg/m^2^]. In addition, BMI-for-age percentiles (pBMI) by sex were calculated by consulting the tables for children and adolescents 2 to 20 years of age provided by the CDC (Centers for Disease Control and Prevention), which included the following criteria: >5th percentile indicates underweight, 5th–85th percentile indicates healthy weight, 85th–94th percentile indicates overweight, ≥95th percentile indicates obesity (15).

#### 2.4.2. Blood pressure

Systolic and diastolic blood pressure was measured in the dominant arm of the volunteer while in a seated position using a Welch Allyn® Baumanometer according to clinical guidelines (16, 17).

#### 2.4.3. Biochemical measurements

Venous blood samples were collected from the forearm after a 12-hour fast according to standard techniques. The samples were collected in the educational centers and later transported in a rigid thermal container with cold packs to a laboratory where the blood serum was separated by centrifugation. Subsequently, the serum samples were analyzed in a single central laboratory in the capital of San Luis Potosí. Total cholesterol, low-density lipoprotein (LDL) cholesterol, high-density lipoprotein (HDL) cholesterol, triglyceride, and glucose levels were assessed on the automated analytical platform BS300 (Mindray®, Nanshan, Shenzhen, China) following the internal test protocols and the use of commercially available reagent kits. For the classification of hyperglycemia, levels greater than 100 mg/dl (5.55 mmol/L) were considered the clinical cutoff point, while for prediabetes, levels of ≥100 mg/dl (5.55 mmol/L) to <126 mg/dl (6.99 mmol/L) were considered the cutoff point according to the standards established by international guidelines (18).

### 2.5. Statistical analysis

Measures such as frequencies and percentages were obtained for categorical variables, while for continuous variables, the mean, standard deviation, median and minimum and maximum intervals were obtained. The Kolmogorov–Smirnov test was used to examine the normality of the data.

The bivariate analysis to evaluate the association between the categorical variables (sex and exercise pattern) and glycemia was performed using Pearson's chi-square statistic (*X*^2^). In addition, the odds ratio (OR) with 95% confidence interval was calculated using a 2 × 2 contingency table, and the association of sociodemographic, lifestyle and cardiometabolic variables with blood glucose was analyzed by Spearman correlation analysis. The sum of hours of video game, cell phone and television use was also included. The results for which *p* < 0.05 were considered statistically significant. Only significantly related variables from the bivariate analysis were included in the multiple linear regression analysis.

The assumptions of the linear regression model were considered in the analysis; for example, to avoid multicollinearity among the variables, a correlation coefficient of *r* < 0.300, *p* < 0.05 and a variance inflation factor (VIF) < 4 were considered. Finally, 4 models with these characteristics were built. The statistical software used was SPSS, version 20.0 (SPSS Inc., Chicago, IL, USA).

## 3. Results

A total of 642 participants were evaluated, of whom 350 were female. With respect to socioeconomic status, 61% had medium-low to low socioeconomic status, 24% had minimum practicality and comfort and 37% spent most of their economic income on food, with their households lacking most utilities. Most participants had an adequate sleep period, although 29.7% slept less than 8 h per day (Table 1), and 98% had a diet low in fiber, fruits and vegetables (data not shown).

**Table 1:** Sociodemographic and lifestyle characteristics (*n* = 642).

Variable	Category	*n* or average	% or SD
**Sociodemographics**			
Sex	Males	292	45.9
	Females	350	54.5
**Socioeconomic status** **^a^**			
A/B (>193)	Planning and future	33	5.50
C+ (155–192)	Entertainment and communication	75	12.4
C (128–154)	Practical life	126	20.9
C− (105–127)	Minimum of practicality	146	24.2
D+ (80–104)	Basic sanitary conditions	120	19.9
D (33–79)	Walls and some services	102	16.9
E (0–32)	Shortage	2	0.30
**Lifestyle**			
Time in front of electronic screens (hours)	TV	2.12^b^	±1.43^c^
	Cell phones	2^b^	±2.23^c^
	Video games	0.63^b^	±1.20^c^
	Total screen time	4.67^b^	±3.23^c^
Sleep (hours)	–	8.50^b^	±1.76^c^
Exercise pattern	Adequate	399	62.7
	Inadequate	237	37.3

^a^ Socioeconomic status (scores).

^b^ Data expressed as averages.

^c^ SD, standard deviation.

The values of cardiometabolic variables were found to be within the reference range (Table 2).

**Table 2:** Characteristics of cardiometabolic factors.

Variable	Average	SD	Median	Minimum-maximum
**Total (*n* = 642)**				
Glucose^a^	89.7 (4.98)	±10.7 (0.59)	89 (4.94)	67–283 (3.72–15.7)
BMI^b^	20.4	±4.5	19.7	12.9–38.2
Systolic BP^c^	114	±12.9	114	72–162
Diastolic BP^c^	65.1	±9.2	65	40–108
Total Cholesterol^a^	150 (3.88)	±29.6 (0.77)	148 (3.83)	27–276 (0.69–7.14)
HDL Cholesterol^a^	54.4 (1.41)	±11.5 (0.30)	53.2 (1.38)	5.4–110 (0.14–2.85)
LDL Cholesterol^a^	76.4 (1.98)	±24.9 (0.64)	74.1 (1.92)	15.9–199 (0.41–5.15)
Triglycerides^a^	98.2 (1.11)	±54.7 (0.62)	84 (0.95)	23–608 (0.26–6.86)
**Females (*n* = 350)**				
Glucose^a^	88.5 (4.92)	±12.7 (0.70)	88 (4.89)	67–283 (3.72–15.7)
BMI^b^	20.2	±4.1	19.9	12.9–35.9
Systolic BP^c^	113	±11.5	113	72–150
Diastolic BP^c^	65.2	±8.7	66	43–108
Total Cholesterol^a^	154 (3.98)	±29.9 (0.77)	152 (3.93)	27–276 (0.69–7.14)
HDL Cholesterol^a^	53.3 (1.38)	±11.4 (0.30)	52.1 (1.35)	5.4–91.5 (0.14–2.37)
LDL Cholesterol^a^	80 (2.07)	±24.5 (0.63)	77.8 (2.01)	15.9–199 (0.41–5.15)
Triglycerides^a^	103 (1.16)	±52.4 (0.59)	87 (0.98)	23–333 (0.26–3.76)
**Males (*n* = 292)**				
Glucose^a^	90.8 (5.04)	±7.4 (0.41)	90.5 (5.03)	70–114 (3.89–6.33)
BMI^b^	19.9	±4.9	18.5	13.2–38.2
Systolic BP^c^	115	±14	115	78–162
Diastolic BP^c^	64	±9.5	64	40–105
Total Cholesterol^a^	153 (3.96)	±27 (0.70)	152 (3.93)	86–240 (2.22–6.21)
HDL Cholesterol^a^	54.9 (1.42)	±11.7 (0.30)	53.6 (1.39)	29.9–110 (0.77–2.85)
LDL Cholesterol^a^	79.9 (2.07)	±22.3 (0.58)	77.9 (2.01)	22–155 (0.57–4.01)
Triglycerides^a^	90.9 (1.03)	±50.3 (0.57)	78 (0.88)	23–608 (0.26–6.86)

^a^ Units expressed in mg/dl and (mmol/L).

^b^ BMI, body mass index (kg/m^2^).

^c^ BP, blood pressure (mmHg); SD, standard deviation.

The pBMI was also analyzed by percentiles for age and sex (male and female) as follows: 5th percentile (14.1 kg/m^2^ and 14.6 kg/m^2^), 50th percentile (19.3 kg/m^2^ and 20.3 kg/m^2^) and 85th percentile (25.3 kg/m^2^ and 25 kg/m^2^). Regarding this, 85% of the participants had a normal BMI or a BMI less than 25 kg/m^2^, while 15% were overweight and obese.

The prevalence of hyperglycemia in males and females was 8.2%, while that of prediabetes was 8% for both sexes. A significant relationship was found between male sex and hyperglycemia (*X*^2^ = 11.7, *p* = 0.001), where it was observed that males were 2.7 times more likely to develop hyperglycemia than females (OR: 2.7, 95% CI: 1.5–5.0). Regarding correlations between glucose and the other variables, systolic blood pressure was positively correlated with glucose levels (*r* = 0.347, *p* < 0.01) in males, and it was the strongest correlation in this study. Additionally, it was observed that socioeconomic status, time spent in front of video game screens, BMI, blood pressure, total cholesterol, LDL cholesterol, triglyceride levels, and an unhealthy diet were positively correlated with glucose levels in both sexes, and most of these findings were retained when analyzed by sex (Table 3).

**Table 3:** Spearman correlation of factors with glucose levels.

	AGE	SES	HS	TV	TCP	TVG	TES	HEI	BMI	SBP	DBP	TC	HDL	LDL	TG
**Total (*n* = 642)**															
GLU	0.071	0.187**	−0.019	0.000	0.014	0.146**	0.056	−0.101*	0.107**	0.305**	0.253**	0.222**	0.015	0.215**	0.113**
**Females (*n* = 350)**															
GLU	0.080	0.125*	−0.030	0.018	0.006	0.039	0.009	−0.082	0.038	0.250**	0.240**	0.228**	0.074	0.185**	0.111*
**Males (*n* = 292)**															
GLU	0.117*	0.246*	−0.040	0.019	0.068	0.152**	0.123*	−0.109	0.226**	0.347**	0.310**	0.221**	−0.077	0.253**	0.167**

SES, socioeconomic status; HS, hours of sleep; TV, time spent in hours in front of a TV; TCP, time spent in hours in front of a cell phone; TVG, time spent in hours in front of video game screens; TES, sum of the total time in hours spent in front of electronic screens; HEI, healthy eating index; BMI, body mass index; SBP, systolic blood pressure; DBP, diastolic blood pressure; TC, total cholesterol; HDL, high-density lipoprotein cholesterol; LDL, low-density lipoprotein cholesterol; TGs, triglycerides; GLU, glucose.

* Correlation is significant, *p *< 0.05.

** Correlation is significant, *p *< 0.01.

From the results obtained from the multiple models (Table 4), it was observed that the factors that were associated with variability in serum glucose concentrations were male sex, socioeconomic status, systolic and diastolic blood pressure, and total cholesterol, LDL cholesterol and triglyceride levels. Particularly, Model 2 presented the greatest explanation of the variability in glucose concentrations, with 15.6% (*p *< 0.05) of the variability explained when combining variables such as sex, socioeconomic status, time spent in front of video game screens, the healthy eating index, BMI, diastolic blood pressure and total cholesterol level.

**Table 4:** Final models of multiple linear regression analysis for glycemia.

Model	Independent variable	Coefficient (*β*)		SD	*p*	R^2^
		Not standardized	Standardized			
1	Systolic blood pressure	0.189	0.230	0.032	<0.001*	0.108
	LDL	0.094	0.208	0.017	<0.001*	
	Triglycerides	0.004	0.018	0.008	0.637	
	Sex (Male vs. Female)	1.848	0.086	0.819	0.024*	
2	Video game time	0.156	0.025	0.257	0.545	0.156
	BMI	0.052	0.031	0.071	0.463	
	Diastolic pressure	0.188	0.221	0.035	<0.001*	
	Total cholesterol	0.041	0.153	0.011	<0.001*	
	SES	0.027	0.140	0.008	0.001*	
	HEI	−0.062	−0.071	0.035	0.079	
	Sex (Male vs. Female)	3.272	0.209	0.648	<0.001*	
3	Video game time	0.089	0.014	0.260	0.731	0.135
	BMI	0.069	0.041	0.072	0.338	
	Diastolic blood pressure	0.202	0.237	0.035	<0.001*	
	SES	0.030	0.155	0.008	<0.001*	
	HEI	−0.063	−0.072	0.036	0.077	
	Sex (Male vs. Female)	3.310	0.212	0.656	<0.001*	
4	Video game time	0.142	0.023	0.261	0.588	0.116
	SES	0.031	0.157	0.008	<0.001*	
	HEI	−0.088	−0.100	0.036	0.015*	
	LDL	0.053	0.159	0.014	<0.001*	
	Triglycerides	0.014	0.091	0.006	0.028*	
	Sex (Male vs. Female)	3.314	0.212	0.665	<0.001*	

BMI, Body mass index; SES, Socioeconomic status; HEI, Healthy eating index; LDL, Low density lipoprotein cholesterol; SD, Standard deviation.

* Significant relationship, *p *< 0.05.

This shows that, for example, male sex is associated with an increase in blood glucose levels of 3.27 mg/dl (0.181 mmol/L) while having a high socioeconomic status can increase glucose levels by 0.027 mg/dl (0.001 mmol/L). In addition, for every 1 mmHg increase in diastolic blood pressure, glucose increased by 0.188 mg/dl (0.001 mmol/L), and for every 1 mg/dl (0.026 mmol/L) increase in total cholesterol, there was a 0.041 mg/dl (0.002 mmol/L) increase in glucose.

When the final models were analyzed by sex (Table 5), it was observed that for males, the greatest amount of variation in glucose concentrations (17.3%, *p* < 0.05) was explained by the combination of time spent in front of video game screens, socioeconomic status, LDL cholesterol level, BMI, and diastolic blood pressure, which were very similar in the other models.

**Table 5:** Final models of multiple linear regression analysis for glycemia by sex.

Sex	Model	Independent variable	Coefficient (*β*)		SD	*p*	R^2^
			Not standardized	Standardized			
Males	1	Age	0.202	0.092	0.130	0.122	0.086
		Total cholesterol	0.046	0.167	0.016	0.004*	
		Video game time	0.179	0.035	0.301	0.553	
		SES	0.039	0.205	0.011	0.001*	
	2	LDL	0.053	0.154	0.020	0.009*	0.095
		BMI	0.172	0.112	0.092	0.063	
		Video game time	0.122	0.024	0.298	0.683	
		SES	0.038	0.203	0.011	0.001*	
	3	Video game time	0.073	0.014	0.287	0.800	0.173
		SES	0.039	0.208	0.011	<0.001*	
		LDL	0.049	0.143	0.020	0.012*	
		BMI	0.070	0.046	0.091	0.443	
		Diastolic blood pressure	0.225	0.285	0.045	< 0.001*	
	4	SES	0.035	0.187	0.011	0.001*	0.152
		Systolic blood pressure	0.166	0.317	0.032	<0.001*	
		Total screen time	0.001	0.001	0.129	0.991	
		Triglycerides	0.002	0.013	0.007	0.824	
Females	1	Systolic blood pressure	0.207	0.181	0.061	0.001*	0.083
		Total cholesterol	0.099	0.228	0.023	<0.001*	
		SES	0.014	0.044	0.018	0.412	
	2	SES	0.013	0.038	0.017	0.473	0.092
		Diastolic blood pressure	0.326	0.219	0.080	<0.001*	
		LDL	0.110	0.208	0.029	<0.001*	
		Triglycerides	0.004	0.015	0.014	0.782	
	3	SES	0.016	0.049	0.018	0.360	0.084
		LDL	0.125	0.237	0.029	<0.001*	
		Triglycerides	−0.002	−0.008	0.014	0.887	
		Systolic blood pressure	0.232	0.202	0.063	<0.001*	
	4	SES	0.011	0.034	0.017	0.526	0.094
		Diastolic blood pressure	0.315	0.211	0.080	<0.001*	
		Total cholesterol	0.091	0.210	0.023	<0.001*	

BMI, Body mass index; SES, Socioeconomic status; LDL, Low density lipoprotein; SD, Standard deviation.

* Significant relationship, *p *< 0.05.

However, for females, Model 4 presented the greatest explanation in the variability of glucose levels (9.4%, *p* < 0.05) when combining socioeconomic status, diastolic blood pressure and total cholesterol level. In general, the variables associated with increased glucose in females were blood pressure, some cardiometabolic variables, and socioeconomic status.

## 4. Discussion

Hyperglycemia has been related to cardiometabolic diseases and can manifest without clear symptoms. Therefore, its identification is clinically important, especially in this population, because children and adolescents are presenting with hyperglycemia or even prediabetes and lipid alterations, all of which increase the risk of T2D and cardiovascular complications in adulthood. As mentioned above, this study focused on evaluating the association of sociodemographic, lifestyle, and cardiometabolic factors with serum glucose levels in children and adolescents in Mexico.

The prevalence of hyperglycemia in our study sample was 8.2%, and the factors that were associated with higher blood glucose levels were male sex, high socioeconomic status, excessive use of video games, inadequate diet, increased BMI, elevated blood pressure, and increased total cholesterol, LDL cholesterol and triglyceride levels. These differences were notable in males, who were more than twice as likely to have hyperglycemia as females.

The mean glucose level was 89.7 mg/dl (4.98 mmol/L), slightly higher than that reported in another study with Mexican adolescents: 80.6 mg/dl (4.47 mmol/L) (19). These results are within the normal clinical range for glucose levels (18); however, it has been reported that even fasting blood glucose levels of 86–99 mg/dl (4.77–5.49 mmol/L) are high enough to double the risk of developing prediabetes and T2D in adulthood (20). Moreover, in our study, 8% of the participants had hyperglycemia and prediabetes. In Mexico, the prevalence of prediabetes reported in a study conducted with children and adolescents in the state of Yucatan, Mexico, was 13% (9). In another study of 2,117 Mexican children and adolescents, the prevalence of prediabetes was 11.3% (21). However, the prevalence of hyperglycemia, although relatively low, could be the result of early-onset IR, which is alarming because without early detection, these children and adolescents are at greater risk of developing T2D. Furthermore, children and adolescents with prediabetes in turn have other comorbidities, such as IR, overweight, obesity and prehypertension, that increase the risk of cardiovascular disease in the future (5, 6).

Regarding sex, similar to our results, other authors have reported that male adolescents have higher glucose levels than females (22, 23). In this context, it has been suggested that the possible underlying mechanism by which males have elevated glucose levels may be related to growth and multiple hormonal changes (24).

Although we did not classify this study population into prepubertal, pubertal and postpubertal groups, these different stages of development have been shown to be indicative of age and hormone production; for example, growth hormone is involved in glycogen metabolism (i.e., glycogenolysis and gluconeogenesis) and lipid metabolism throughout childhood. In addition, it has been reported that at prepubertal ages, there is a greater preferential oxidation of fat over carbohydrates during exercise, in contrast to pubertal and postpubertal subjects (25).

Another variable that we analyzed was socioeconomic status; in this case, our results indicated that higher socioeconomic conditions were positively associated with glycemia. However, the results of other studies are still inconsistent regarding the exact relationship between socioeconomic status and the development of metabolic diseases; for example, in a cohort study, it was shown that low socioeconomic conditions are associated with the development of prediabetes in children and adolescents (26, 27). Conversely, children from families of higher socioeconomic status have been reported to have more than twice the risk of developing prediabetes (28). This may be because sedentary behaviors, such as having access to TVs and video games or to private transportation and thus not having to walk from one place to another, may be characteristic of the lifestyles of families with higher socioeconomic status.

Similar to the results of this study, the excessive use of video games (>5 h a day) combined with low physical activity (<1 h a day) was associated with higher glucose (*β* > 0.100, *p* < 0.05) in adolescents (29). In our study, the average time spent using electronic screens was >4 h a day, and most of the participants did not exercise. Excess time dedicated to video games and physical inactivity are indicative of a sedentary lifestyle and therefore would be associated with higher glucose levels. In fact, one study reported that higher glucose levels were associated with sedentary lifestyles in healthy adults (30). Although we did not evaluate sedentary behavior, the evidence shows that a sedentary lifestyle influences the variability of glucose levels (31).

Regarding dietary factors, a healthy diet was associated with lower glucose levels in this study, which is consistent with other studies where adequate nutrition had a positive impact on health in both adults and adolescents (32, 33).

Unhealthy diets, such as the Western diet, are characterized by high consumption of refined sugars, animal fats, processed meats, and refined cereals with low intake of fiber, fruits and vegetables (34), which was characteristic of our population. Foods such as fiber, fruits and vegetables have beneficial effects (35); for example, some foods contain components such as anthocyanins that could lower insulin resistance and thereby reduce the risk of T2D by inhibiting the enzymatic action of α-amylase and α-glucosidase and glucose transporters (GLUT) (36, 37).

Our results showed an association between high BMI and high glucose concentrations. These results were expected and are consistent with those previously reported in individuals aged less than 22 years (38, 39). For example, a positive correlation was found between BMI and glucose in children and adolescents in both sexes (*r* = 0.124, *p* = 0.014) (40). The same relationship was noted in the present study (*r* = 0.107, *p* < 0.001); however, in both cases, the correlation was weak. In addition, this study revealed a positive correlational trend between BMI and glucose in male subjects (*r* = 0.148, *p *= 0.052), which was higher than that of female subjects. A previous study revealed the same correlation between BMI and glucose, also with a stronger trend in men (*r* = 0.226, *p *< 0.001) than in women (*r* = 0.038, *p* > 0.05), highlighting a higher probability that a high BMI in adolescence is associated with a state of hyperglycemia in adulthood (41).

Another finding of this study was that high blood pressure values were associated with high glucose levels. A positive relationship has been reported between these two cardiometabolic variables in children and adolescents between 7 and 17 years of age (42); moreover, adolescents with high glucose levels are more likely to have hypertension (OR: 4.6; 95% CI: 1.6–12.7) (43). In fact, one study showed that when evaluating a group of adolescents with serum glucose levels higher than 100 mg/dl (5.55 mmol/L), they had alterations in systolic blood pressure (44), which emphasizes the evaluation of glucose levels from an early age to prevent future cardiovascular diseases. Fasting hyperglycemia may be associated with arterial stiffness, possibly caused by oxidative stress, the accumulation of glycation end products, and alterations in vasoactive substance mechanisms (45). In addition, the synergistic effects of blood glucose and blood pressure levels, even at levels below the clinical values that define T2D and hypertension, can lead to the progression of arteriosclerotic arterial damage (46).

The positive association between total cholesterol, LDL cholesterol and triglyceride levels with glucose is similar to other results reported in adults (47, 48) and in normal-weight children, who for example, were shown to have an association between dyslipidemia and impaired fasting glucose (49). Impaired fasting glucose may be caused by impaired free fatty acid metabolism due to the induction of IR underlying the lipid abnormality, which triggers the hepatic overproduction of glucose, resulting in elevated blood glucose levels (49). In addition, it has been reported that the development of IR is related to a decrease in HDL and an increase in LDL in children and adolescents of both sexes (50).

The limitations of this study were that it was a cross-sectional study, so it is not possible to determine causality, and the use of the questionnaires constructed for this study limits the scope of detection. In addition, in this study, we evaluated the elementary and high school populations because they are vulnerable populations and are thus understudied; however, subgroup analyses were not performed for the different stages of maturation (i.e., prepubertal, pubescent and postpubertal). Additionally, we know that most of these factors require previous clinical or biochemical tests and therefore would be a limiting factor for the early identification of hyperglycemia. Finally, another limitation of this study was that more socioecological factors were not analyzed, which are important to consider, as they may provide a more holistic approach to the multiple interrelated factors that may influence or affect people's health status when assessing different individual, interpersonal, community, organizational and political factors.

Nevertheless, one of the strengths of our study is that we explored the association of sociodemographic, lifestyle and cardiometabolic factors, which in turn are individual and intrapersonal factors of the socioecological model, in young Mexican students attending public institutions; although this study is not representative of the entire Mexican population, there are no studies that integrate these factors and their association with glycemia in children and adolescents from San Luis Potosí, Mexico. Furthermore, we included an adequate sample size to generate sufficient statistical power. Finally, the importance of evaluating differing lifestyles in addition to clinical biomarkers as important factors related to glycemia and the increasing emphasis on primary care should be highlighted since the presence of hyperglycemia in children and adolescents may increase the risk of NCDs in adulthood.

In the future, it is important to continue investigating the interaction of more biochemical markers stratified by age subgroups as well as other factors of the socioecological model at the individual (e.g., race, genetics, parental education), interpersonal (e.g., number of family members), organizational (e.g., work environment), community and political levels, which would provide a complete and explanatory panorama of this phenomenon to help implement effective health interventions.

## 5. Conclusion

Several sociodemographic, lifestyle, and cardiometabolic factors are positively associated with glucose levels in Mexican children and adolescents.

Finally, the identification of each of these risk factors in young populations can be useful in the development of future preventive strategies to reduce the risk of developing NCDs in the medium or long term as well as in decision-making in public health to improve the quality of life of children and adolescents.

## Data Availability

The original contributions presented in the study are included in the article, further inquiries can be directed to the corresponding author/s.
